# Supplementary material for: DNA methylation at the suppressor of cytokine signaling 3 (SOCS3) gene influences height in childhood
Source: Nat Commun. 2023 Aug 25;14:5200. doi: 10.1038/s41467-023-40607-0 (PMC10457295; doi:10.1038/s41467-023-40607-0)
Supplement: Supplementary file 1 — Supplementary Information [file 41467_2023_40607_MOESM1_ESM.pdf]

Supplementary Information

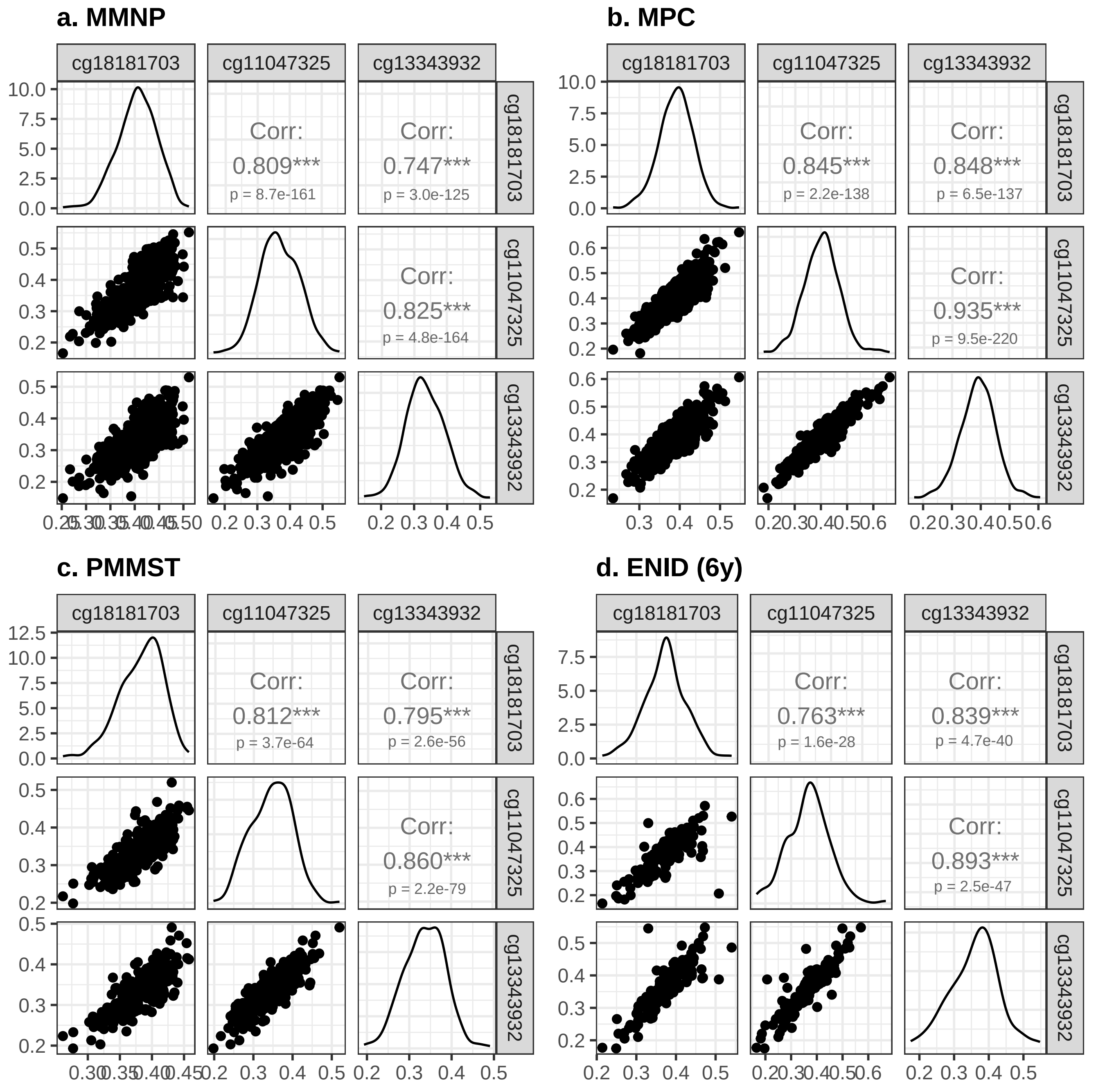

Supplementary Figure 1: Correlation of methylation between three SOCS3 CpGs: a) MMNP; b) MPC; c) PMMST; and d) ENID at 6 years. In these cohorts, methylation was measured using EPIC Infinium Methylation array. For a-d, Pearson correlation coefficient (r) and p-value (using corr.test() in R) is shown. Corr: Pearson's correlation coefficient. MMNP: Mumbai Maternal Nutrition Project; MPC: Mysore Parthenon Cohort; PMMST: Peri-conceptional Multiple Micronutrients Supplementation Trial; ENID: Early Nutrition and Immune Development.

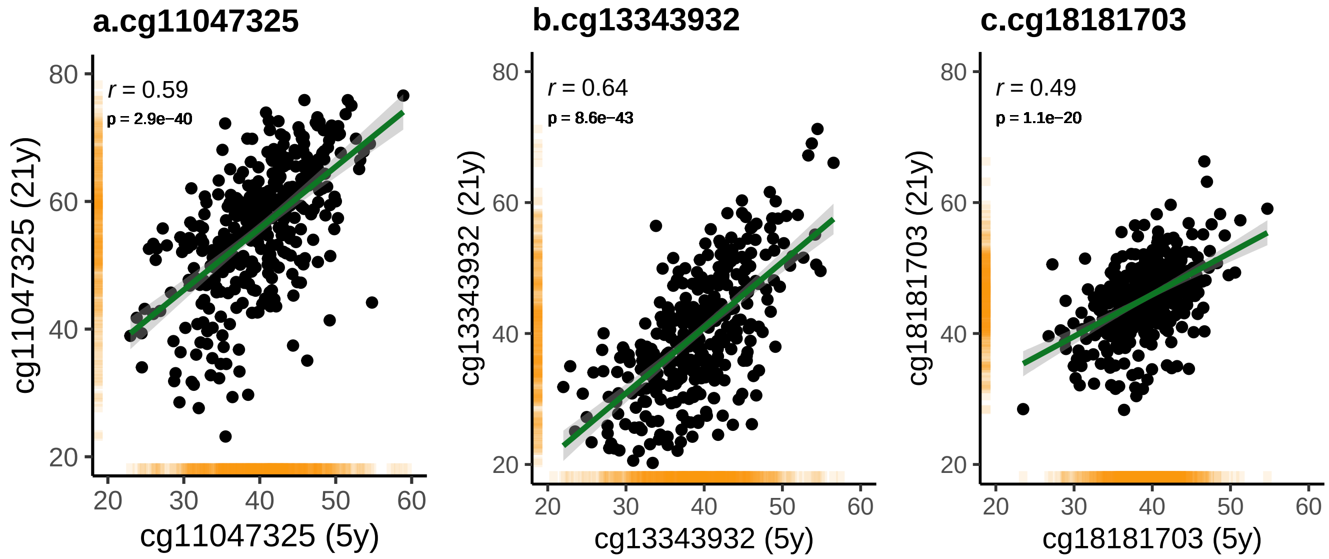

Supplementary Figure 2: Stability of SOCS3 methylation between 5 and 21 years. Scatter plots show correlation of methylation at the three SOCS3 CpGs between 5 years (EPIC data) and 21 years (pyrosequencing data) in the MPC cohort. For a-c, Pearson correlation coefficient (r) and p-value (using corr.test() in R) is shown. The shaded areas around the regression lines indicate 95% confidence interval. r: Pearson's correlation coefficient. MPC: Mysore Parthenon Cohort.

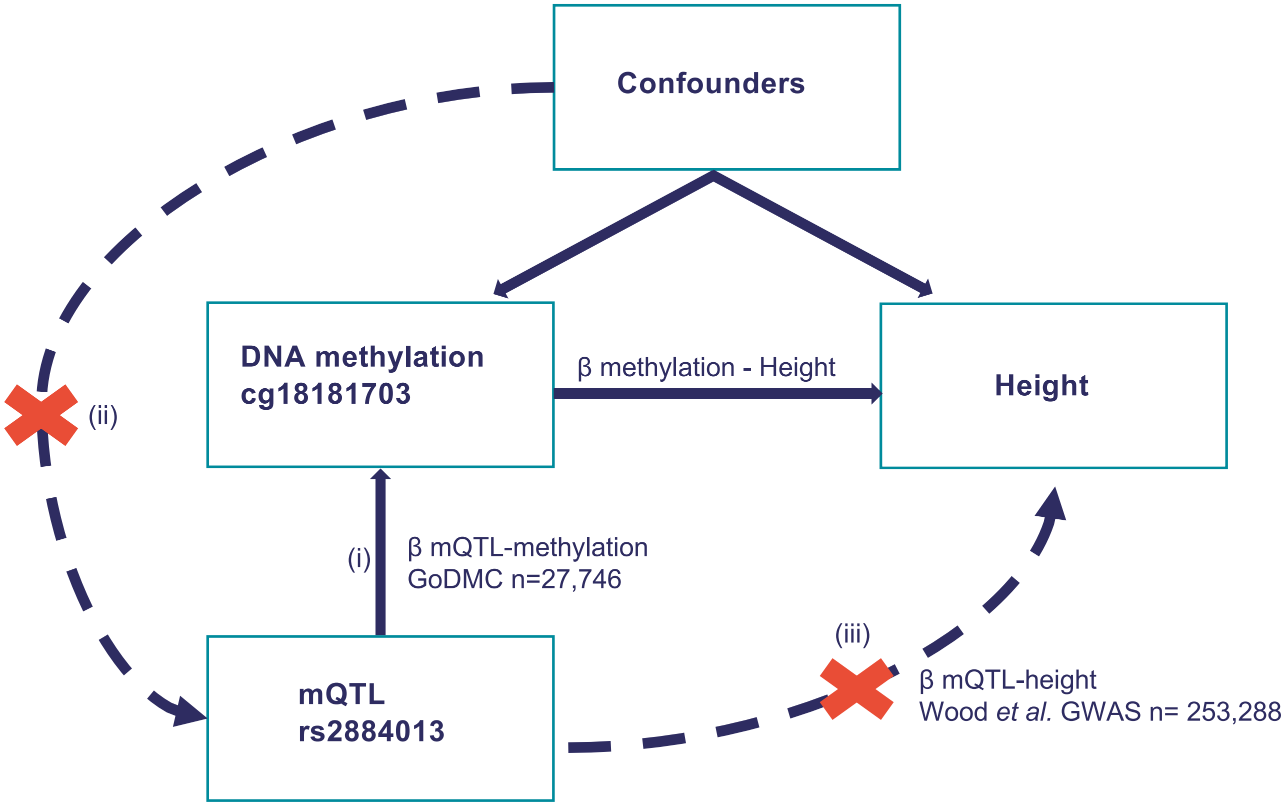

Supplementary Figure 3: Two Sample Mendelian Randomization analysis investigating the causal effect of *SOCS3* methylation on height. MR was carried out with DNA methylation as exposure and height as an outcome with the assumptions that i) there is a strong association between the genetic variant and the exposure ii) the genetic variant is not associated with any of confounders of the exposure-outcome association and iii) the genetic variant is associated with the outcome via the exposure only. rs2884013 identified as an mQTL for cg18181703 from GoDMC data (n = 27,746) was used as an instrumental variable. Strength of association between the mQTL and height was estimated using summary data from a large height GWAS (n = 253,288). The causal effect was quantified by Wald ratio estimator (see Methods). MR: Mendelian Randomization, mQTL : methylation Quantitative trait loci, β: effect size.

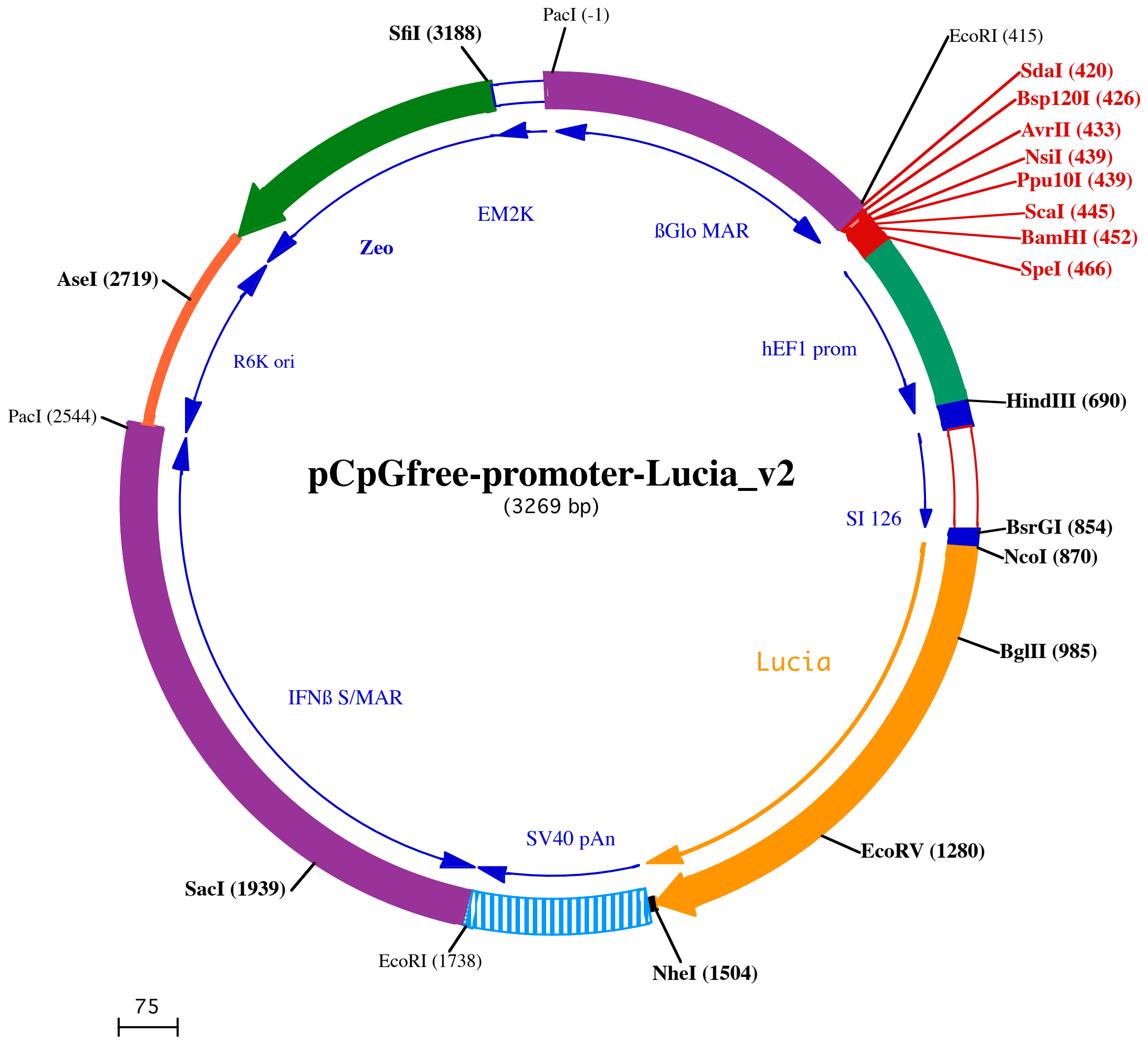

Supplementary Figure 4: Vector map of pCpGfree-promoter-Lucia: SOCS3 fragments were cloned into multiple cloning sites (MCS) ahead of the minimal promoter (hEF1 prom) using ApaI and BamHI restriction enzymes. The size of the vector is 3269 bp with Zeocin based selection and Lucia (secreted) based reporter assay system. The vector harbours no CpG dinucleotides hence eliminates the possibility of spurious effects from methylation of vector backbone which makes it suitable for in vitro methylation of the recombinant plasmid with cloned fragments and direct correlation of reporter gene expression to methylation of the (SOCS3) inserts. Description of the plasmid features can be accessed from Catalog: pcpgf-promlc (https://www.invivogen.com/)

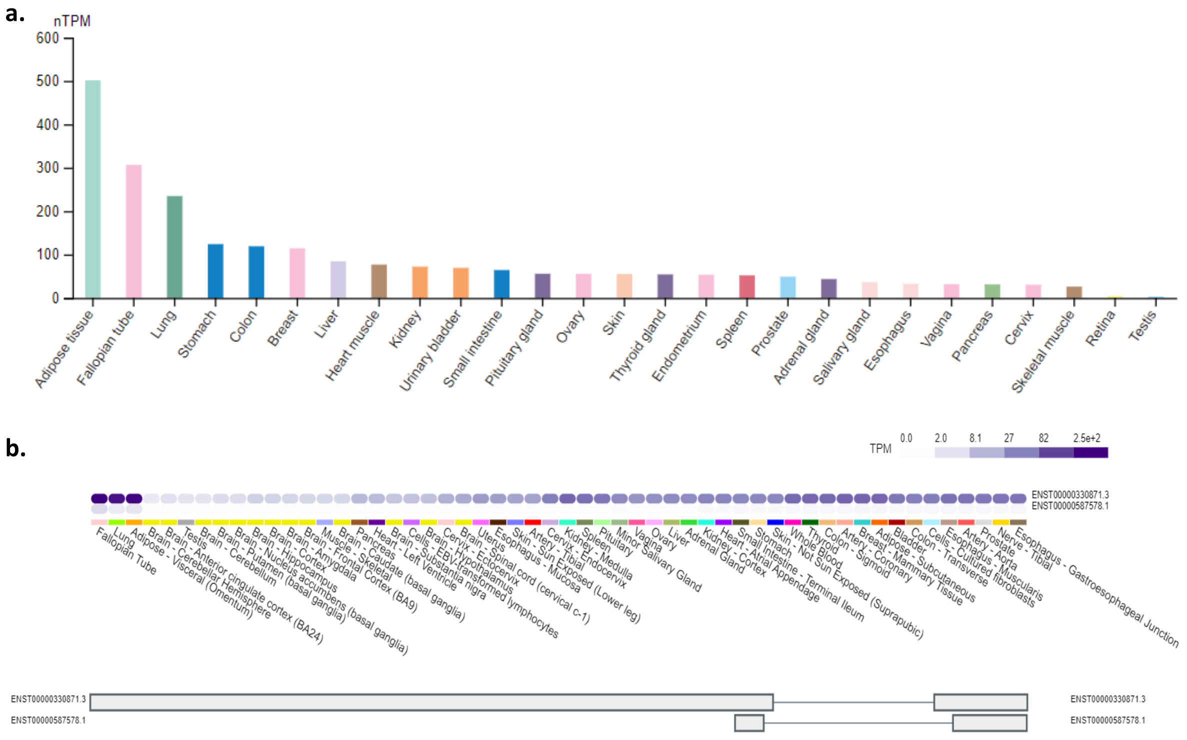

Supplementary Figure 5: SOCS3 gene expression profile a) for different tissues from Gene -Tissue expression (GTEx). Y-axis: number of transcripts per million (nTPM). b) SOCS3 gene isoforms expression across different tissues [Source: HGNC Symbol; Acc: HGNC:19391]. For a and b, data Source: GTEx Analysis Release V8 (dbGaP Accession phs000424.v8.p2; https://gtexportal.org/home/).
