# Supplementary material for: DNA methylation at the suppressor of cytokine signaling 3 (SOCS3) gene influences height in childhood
Source: Nat Commun. 2023 Aug 25;14:5200. doi: 10.1038/s41467-023-40607-0 (PMC10457295; doi:10.1038/s41467-023-40607-0)
Supplement: Supplementary file 3 — Description of Additional Supplementary Files [file 41467_2023_40607_MOESM3_ESM.pdf]

Description of Additional Supplementary Files

Supplementary Data 1: Height EWAS results adjusted for blood cell counts

Supplementary Data 2: Sensitivity analysis for the supplementation group in the EMPHASIS cohorts

Supplementary Data 3: Differentially methylated region (DMR) analysis results.

Supplementary Data 4. Association of EWAS significant loci in replication cohorts

Supplementary Data 5: Sensitivity analysis for seasonality in date of conception in the Gambian cohorts

Supplementary Data 6: Summary of cis-mQTL analysis for three *SOCS3* CpGs

Supplementary Data 7: Meta-analysis results of *SOCS3m* - SNP associations

Supplementary Data 8: Sensitivity analysis for *SOCS3* methylation -height associations adjusted for height PRS in the replication cohorts.

Supplementary Data 9: Proportion of height variance explained by *SOCS3* methylation and height PRS

Supplementary Data 10: Comparison of variance explained by *SOCS3* methylation and PRS from birth to adulthood in MPC

Supplementary Data 11: Longitudinal height analysis in the MPC cohort

Supplementary Data 12: Conditional height analysis in the MPC cohort

Supplementary Data 13: Mendelian Randomization (MR) results for causal effect of *SOCS3* methylation on height

Supplementary Data 14: Summary statistics of prenatal exposure measures in the study cohorts

Supplementary Data 15: Association analysis between prenatal maternal exposures and *SOCS3* methylation.

Supplementary Data 16: Effect of maternal folate and SES on *SOCS3* methylation

Supplementary Data 17: Meta-analysis of SNPs associated with serum folate in Indians for selecting folate instrumental variable

Supplementary Data 18: Linkage disequilibrium between the four folate associated SNPs in South Asian Population in 1000Genome Phase3

Supplementary Data 19: Association of maternal folate SNP genotypes (instrument) with child CpG methylation (outcome)

Supplementary Data 20: Mendelian Randomization analysis of maternal folate (during pregnancy) and CpG methylation in children

Supplementary Data 21: Results of association analysis between *SOCS3* methylation and BMI at 5 and 21 years (MPC)

Supplementary Data 22: Pyrosequencing assay details

Supplementary Data 23: Oligo primer sequences used for cloning *SOCS3* region
