# Supplementary material for: DNA methylation at the suppressor of cytokine signaling 3 (SOCS3) gene influences height in childhood
Source: Nat Commun. 2023 Aug 25;14:5200. doi: 10.1038/s41467-023-40607-0 (PMC10457295; doi:10.1038/s41467-023-40607-0)
Supplement: Supplementary file 27 — Reporting Summary [file 41467_2023_40607_MOESM27_ESM.pdf]

nature portfolio

Corresponding author(s): Matt J. Silver (matt.silver@lshtm.ac.uk)
Giriraj R. Chandak (chandakgrc@ccmb.res.i

Last updated by author(s): Jun 23, 2023

Reporting Summary

Nature Portfolio wishes to improve the reproducibility of the work that we publish. This form provides structure for consistency and transparency in reporting. For further information on Nature Portfolio policies, see our Editorial Policies and the Editorial Policy Checklist.

Statistics

For all statistical analyses, confirm that the following items are present in the figure legend, table legend, main text, or Methods section.

n/a	Confirmed	
☐	☒	The exact sample size (*n*) for each experimental group/condition, given as a discrete number and unit of measurement
☐	☒	A statement on whether measurements were taken from distinct samples or whether the same sample was measured repeatedly
☐	☒	The statistical test(s) used AND whether they are one- or two-sided<br>*Only common tests should be described solely by name; describe more complex techniques in the Methods section.*
☐	☒	A description of all covariates tested
☐	☒	A description of any assumptions or corrections, such as tests of normality and adjustment for multiple comparisons
☐	☒	A full description of the statistical parameters including central tendency (e.g. means) or other basic estimates (e.g. regression coefficient) AND variation (e.g. standard deviation) or associated estimates of uncertainty (e.g. confidence intervals)
☐	☒	For null hypothesis testing, the test statistic (e.g. *F*, *t*, *r*) with confidence intervals, effect sizes, degrees of freedom and *P* value noted<br>*Give P values as exact values whenever suitable.*
☒	☐	For Bayesian analysis, information on the choice of priors and Markov chain Monte Carlo settings
☒	☐	For hierarchical and complex designs, identification of the appropriate level for tests and full reporting of outcomes
☐	☒	Estimates of effect sizes (e.g. Cohen's *d*, Pearson's *r*), indicating how they were calculated

Our web collection on statistics for biologists contains articles on many of the points above.

Software and code

Policy information about availability of computer code

Data collection	No software was used for data collection.
Data analysis	All analyses used open-source software.<br>Software used in this study: R (Version 4.1.2), PLINK (version 1.90b6.24; v2.00a3.3LM), IMPUTE2 (version 2.3.2), SHAPEIT (version 4.2.2), METAL (version release 2011-03-25), zscorer (version 0.3.1), meffil (version 1.3.6), DMRcate (version 2.14.0), GEM (version 1.10.0), and TwoSampleMR (version 0.5.6)

For manuscripts utilizing custom algorithms or software that are central to the research but not yet described in published literature, software must be made available to editors and reviewers. We strongly encourage code deposition in a community repository (e.g. GitHub). See the Nature Portfolio guidelines for submitting code & software for further information.

Data

Policy information about availability of data

All manuscripts must include a data availability statement. This statement should provide the following information, where applicable:

- Accession codes, unique identifiers, or web links for publicly available datasets
- A description of any restrictions on data availability
- For clinical datasets or third party data, please ensure that the statement adheres to our policy

Requests to access the MMNP data should be submitted to K. Kumaran, Giriraj R. Chandak, and for the MPC data to G. V. Krishnaveni. Requests to access the

Gambian data should be submitted to the corresponding authors in the first instance. An application would then need to be made to MRC Unit The Gambia's Scientific Coordinating Committee and the Joint MRC/Gambia Government Ethics Committee. EMPHASIS study data is available on request and will be made publicly available once results from the main EMPHASIS study have been published. ALSPAC data used for this submission is available on request by application to the ALSPAC executive committee (ALSPAC-exec@bristol.ac.uk). The ALSPAC data management plan (available here: www.bristol.ac.uk/alspac/researchers/access/) describes in detail the policy regarding data sharing, which is through a system of managed open access. Source data are provided with this paper. All relevant data supporting the key findings of this study are available within this article and its Supplementary Data files.
Publicly available data used in this study include GoDMC (http://mqtldb.godmc.org.uk/search?query=cg18181703), GWAS catalog (https://www.ebi.ac.uk/gwas/), ENCODE (https://www.encodeproject.org/) and GTex (https://gtexportal.org/home/) databases.

Research involving human participants, their data, or biological material

Policy information about studies with human participants or human data. See also policy information about sex, gender (identity/presentation), and sexual orientation and race, ethnicity and racism.

Reporting on sex and gender	Details of sex distribution for all the study cohorts are described in Table 1. Sex was assigned based on self-report.
Reporting on race, ethnicity, or other socially relevant groupings	Ethnicity was determined based on the geographical location of the participating cohorts.
Population characteristics	Details of participant characteristics from the cohorts analysed in this study are to be found in the relevant published cohort/protocol papers referenced in Methods. Key details relevant to this analysis are presented in Table 1 and in Methods.
Recruitment	This is a secondary analysis of the existing data. Recruitment details for each cohort analysed in this study are to be found in the relevant published cohort/protocol papers referenced in Methods.
Ethics oversight	Ethics approval for the discovery cohort (EMPHASIS - MMNP) was obtained from the Intersystem Biomedical Ethics Committee, Mumbai in 2013 (serial no. ISBEC/NR-54/KM/JVJ/2013). MPC (Indian replication cohort) was approved by Holdsworth Memorial Hospital (HMH) research ethics committee, Mysore, India. Gambian replication cohorts (PMMST and ENID) were approved by the joint Gambia Government / MRC Unit The Gambia Ethics Committee. Ethical approval for the ALSPAC study was obtained from the ALSPAC Ethics and Law Committee and the Local Research Ethics Committees. See 'Ethics and consent' section in the manuscript for further details.

Note that full information on the approval of the study protocol must also be provided in the manuscript.

Field-specific reporting

Please select the one below that is the best fit for your research. If you are not sure, read the appropriate sections before making your selection.

☒ Life sciences ☐ Behavioural & social sciences ☐ Ecological, evolutionary & environmental sciences

For a reference copy of the document with all sections, see nature.com/documents/nr-reporting-summary-flat.pdf

Life sciences study design

All studies must disclose on these points even when the disclosure is negative.

Sample size	This is a secondary data analysis with replication using data collected from five existing cohort studies. Details are provided in the manuscript
Data exclusions	Data filtering and QC for methylation and genotype data for each cohort, along with the rationale for exclusions, is described in Methods. Exclusion criteria were determined prior to conducting the analysis.
Replication	The study follows a discovery-replication design whereby significant loci from the discovery cohort are tested in the replication cohorts. All attempted replications are described in the manuscript.
Randomization	Randomisation not applicable; epigenome-wide analysis of a continous trait
Blinding	Blinding not applicable; epigenome-wide analysis of a continous trait

Reporting for specific materials, systems and methods

We require information from authors about some types of materials, experimental systems and methods used in many studies. Here, indicate whether each material, system or method listed is relevant to your study. If you are not sure if a list item applies to your research, read the appropriate section before selecting a response.

Materials & experimental systems

n/a	Involved in the study
☒	☐ Antibodies
☐	☒ Eukaryotic cell lines
☒	☐ Palaeontology and archaeology
☒	☐ Animals and other organisms
☒	☐ Clinical data
☒	☐ Dual use research of concern
☒	☐ Plants

Methods

n/a	Involved in the study
☒	☐ ChIP-seq
☒	☐ Flow cytometry
☒	☐ MRI-based neuroimaging

Eukaryotic cell lines

Policy information about cell lines and Sex and Gender in Research

Cell line source(s)	All the cell lines used in this study including human lung carcinoma (A549), human embryonic kidney (HEK293T) and human liver carcinoma (HepG2) were obtained from the American Type Culture Collection (ATCC).
Authentication	Cell lines used in this study were authenticated by standard STR profiling method.
Mycoplasma contamination	Cell lines used in this study were tested negative for Mycoplasma contamination.
Commonly misidentified lines (See ICLAC register)	No commonly misidentified cell lines were used.
